# Inflammation and oxidative stress in salt sensitive hypertension; The role of the NLRP3 inflammasome

**DOI:** 10.3389/fphys.2022.1096296

**Published:** 2022-12-22

**Authors:** Lale A. Ertuglu, Ashley Pitzer Mutchler, Justin Yu, Annet Kirabo

**Affiliations:** ^1^ Division of Nephrology, Department of Medicine, Vanderbilt University Medical Center, Nashville, TN, United Staes; ^2^ Division of Clinical Pharmacology, Department of Medicine, Vanderbilt University Medical Center, Nashville, TN, United States; ^3^ Department of Pathology, Microbiology, and Immunology, Vanderbilt University Medical Center, Nashville, TN, United States

**Keywords:** salt sensitivity, hypertension, immunity, inflammation, inflammasome

## Abstract

Salt-sensitivity of blood pressure is an independent risk factor for cardiovascular disease and affects approximately half of the hypertensive population. While the precise mechanisms of salt-sensitivity remain unclear, recent findings on body sodium homeostasis and salt-induced immune cell activation provide new insights into the relationship between high salt intake, inflammation, and hypertension. The immune system, specifically antigen-presenting cells (APCs) and T cells, are directly implicated in salt-induced renal and vascular injury and hypertension. Emerging evidence suggests that oxidative stress and activation of the NLRP3 inflammasome drive high sodium-mediated activation of APCs and T cells and contribute to the development of renal and vascular inflammation and hypertension. In this review, we summarize the recent insights into our understanding of the mechanisms of salt-sensitive hypertension and discuss the role of inflammasome activation as a potential therapeutic target.

## Introduction

Hypertension is among the leading cause of cardiovascular morbidity and mortality, with nearly one-third of the adult population affected globally (Mills et al., 2020). Various environmental and genetic factors are known to associate with high blood pressure, including the dietary sodium intake and the salt-sensitivity of the individuals. While several organ systems including kidneys, vasculature and central nervous system have been implicated in the pathophysiology of salt-sensitivehypertension (Hall et al., 2015), the exact pathophysiological mechanisms underlying its development relationship with sodium remain unclear.

The blood pressure response to sodium is largely dependent on salt-sensitivity. Salt sensitivity of blood pressure is defined as changes in blood pressure that parallel changes in dietary salt intake. More than half of all hypertensive patients and one-fourth of normotensive individuals are affected by salt-sensitivity (Weinberger et al., 1986), which is a risk factor for cardiovascular mortality independent of hypertension (Morimoto et al., 1997; Weinberger et al., 2001). Despite the well-recognized implications of salt-sensitivity in clinical outcomes, its mechanisms are poorly defined. Recent advances in our understanding of body sodium handling have raised the need for revisiting the traditional concepts of salt-sensitivity. Concurrently, mounting evidence introduced immune system as a major factor in the pathogenesis of salt-sensitive hypertension. Indeed, high sodium is a strong stimulus for inflammatory activation and oxidative stress, which leads to vascular and kidney dysfunction resulting in hypertension in experimental settings (Wu et al., 2016).In clinical studies, evidence of inflammation and oxidative stress is consistently linked with elevated blood pressure (Baradaran et al., 2014; Jayedi et al., 2019), and anti-inflammatory treatment with lower blood pressure (Schrader et al., 2007; Sturgis et al., 2009; Brands et al., 2010), as discussed in detail later.

In this review, we will discuss the evidence relating to the roles of inflammation and oxidative stress in salt sensitive hypertension, with an emphasis on the recent findings on inflammasome activation.

## Classical concept and the recent changes in our understanding of salt-sensitive hypertension

The foundations of research on salt-sensitive hypertension was pioneered by Dahl’s seminal work over 60 years ago (Dahl et al., 1962). By mating an unselected strain of Sprague-Dawley rats based on their blood pressure response to salt, Dahl et al. created the first salt-sensitive and salt-resistant rat strains (Dahl et al., 1962). In their later work, Dahl et al. demonstrated that kidney transplantation from a salt-sensitive donor into salt-resistant rat resulted in salt-sensitivity in the recipient; suggesting a major role of renal mechanisms in the development of salt-sensitive hypertension (Dahl and Heine, 1975). Subsequently, the fully inbred strains of Dahl salt-sensitive and salt-resistant rats were developed by Rapp and Dene (Rapp and Dene, 1985) and were used vastly by later animal studies that investigated the pathophysiological basis of salt-sensitivity. A complete discussion of the animal models of salt-sensitivity has been previously provided in Scientific Statement on salt-sensitivity from the American Heart Association (Elijovich et al., 2016).

The classical concept of salt-sensitive hypertension is based on the postulates of Guyton et al. (Guyton, 1991). According to this view, blood pressure homeostasis following an acute salt load is reached by pressure-natriuresis in the kidneys after a salt-induced expansion of plasma volume, which restores baseline levels of blood pressure in healthy individuals (Guyton, 1991). In turn, salt-sensitive blood pressure response was hypothesized to originate from renal Na^+^ excretion dysfunction. While alterations in renal tubular Na^+^ channels (Iwaoka et al., 1991; Lluch et al., 1996; Mu et al., 2011; Shah et al., 2016), renin angiotensin system (Campese et al., 1993; Giner et al., 2000; Poch et al., 2001), and sympathetic system (Fujita et al., 1980; de la Sierra et al., 1996; Ono et al., 1997; Krum et al., 2009) have been shown in salt-sensitive hypertension, hemodynamic studies in humans have later provided evidence that the pathogenesis of salt-sensitivity cannot be explained by pressure-natriuresis. Indeed, hemodynamic changes produced by salt loading and depletion, including renal Na^+^ excretion, balance and plasma volume, do not differ in salt sensitive *versus* resistant individuals (Schmidlin et al., 2007; Laffer et al., 2016). However, salt-sensitivity has been characterized by an absence of the systemic vasodilator response to salt that is found in salt resistance (Sullivan and Ratts, 1983; Schmidlin et al., 2007; Laffer et al., 2016). In a clinical study investigating 24-h hemodynamic changes produced in salt-sensitive *versus* salt-resistant individuals, Laffer et al. (2016) found that salt depletion resulted in an equal reduction in both groups, while a significant increase in total peripheral resistance was only observed in salt resistant individuals. Following salt loading, salt-sensitive individuals were characterized by higher total peripheral resistance and subsequently higher mean arterial pressure compared to salt-resistant individuals. Importantly, recent studies using whole-genome sequencing data from the Trans-Omics in Precision Medicine Whole-Genome Sequencing Program found that variants of SCNN1D, which encodes the δ subunit of ENaC that is poorly expressed in human nephron, is associated with blood pressure as well as estimated glomerular filtration rate, implicating an important role of extrarenal ENaCs in the development of hypertension (Blobner et al., 2022). These findings suggest that pathophysiological mechanisms leading to vascular dysfunction could be a key player in the development of salt-sensitivity.

Our understanding of body salt handling has radically changed in the last 2 decades following the discovery that sodium can be stored in the interstitium of tissue, without commensurate water. Substantial amounts of sodium can be accumulated in the tissue *via* the interaction of these positively charged ions with negatively charged glycosaminoglycans (Titze et al., 2004; Wenstedt et al., 2021). In various subsequent clinical studies, non-osmotic sodium deposition in humans has been shown in the interstitium of skin and skeletal muscle using ^23^Na MRI (Kopp et al., 2013). Importantly, hypertension associates with higher concentrations of sodium deposition (Kopp et al., 2013; Sahinoz et al., 2021a), indicating a potential role of tissue Na^+^ storage in blood pressure regulation and salt-sensitivity (Elijovich et al., 2020; Ertuglu et al., 2021).

## Immune system and salt-sensitive hypertension

The first evidence of a causal relationship between immune activation and hypertension came from experimental models of hypertension showing the blood pressure-lowering effects of immunosuppression (White and Grollman, 1964) and depletion of T-cells *via* neonatal thymectomy (White and Grollman, 1964). Subsequently, fundamental studies by Guzik et al. revealed that T-lymphocytes were essential in the development of hypertension and related vascular dysfunction mice models (Guzik et al., 2007) and cytotoxic CD8^+^ T cells lead to renal vascular remodeling and Na^+^ retention in hypertension (Trott et al., 2014). Furthermore, mice depleted of T cells or interleukin-17A (IL-17A) were protected from the development of endothelial dysfunction and hypertension (Crowley et al., 2010; Madhur et al., 2010). Further studies have shown that depletion of pro-inflammatory cytokines including interleukin-6 (IL-6) (Schrader et al., 2007; Sturgis et al., 2009; Brands et al., 2010) and tumor necrosis factor-alpha (TNF-α) (Guzik et al., 2007; Zhang et al., 2014) ameliorates or prevents hypertension and related end-organ damage. Corroborating with these findings, elevated serum inflammatory markers C-reactive protein, IL-6 and TNF-α correlate with high blood pressure and organ damage in hypertensive patients (Sung et al., 2003; Bautista et al., 2005; Navarro-González et al., 2008; Cheung et al., 2012) and predict the prospective development of hypertensive in non-hypertensives (Sesso et al., 2003; Sesso et al., 2007; Mattace-Raso et al., 2010). The role of immunity in hypertension has been previously discussed elsewhere in detail (Harrison, 2014; Ridker et al., 2017).

Both innate and adaptive immunity play a vital role in the development of hypertension. Antigen-presenting cells (APCs), including primarily dendritic cells (DCs), macrophages, and T cells are the first responders to pro-hypertensive stimuli and activate T-cells through antigen-MHC receptor interaction and co-stimulation (Kambayashi and Laufer, 2014). Antagonism of the B7 ligand (CD80 and CD86) on APCs in mice protects from deoxycorticosterone acetate (DOCA)-salt induced hypertension (Vinh et al., 2010). This immune activation appears to play a crucial role in the kidney-mediated mechanisms of hypertension. High dietary salt leads to renal infiltration of APCs and T-cells and ensuing tissue damage in experimental models of hypertension (Kirabo et al., 2014; Caillon et al., 2017; Pitzer et al., 2020). The extent of immune cell infiltration in the kidneys predicts the degree of blood pressure elevation (Franco et al., 2007; Heijnen et al., 2014). Prevention of renal immune cell infiltration, genetically or pharmacologically, lowers blood pressure and attenuates salt-sensitive hypertension, a finding corroborated by animal studies (Quiroz et al., 2001; Miguel et al., 2010; De Miguel et al., 2011a; De Miguel et al., 2011b; Spradley et al., 2013). Importantly, immunosuppression does not appear to affect blood pressure in normotensive control rat strains (Boesen et al., 2010). Recent studies found that salt sensitive rat models are susceptible to hypertension with high fat diet (Nagae et al., 2009). Spandley et al. demonstrated that salt sensitive Dahl rats on a 4-week high fat diet show significantly more infiltration of T cells in the renal glomerulus and medulla along with more pronounced glomerular injury compared to rats on normal diet. Immunosuppression with mycophenolate mofetil prevented the increase in blood pressure, glomerular immune cell infiltration and injury, but did not alter medullary damage (Spradley et al., 2013).Inflammation is also a key modulator of vascular dysfunction and stiffness seen in hypertension. High sodium results in immune cell infiltration in the vasculature as well as the kidneys (Caillon et al., 2017), which is driven by APC-mediated inflammation in hypertension (De Ciuceis et al., 2005; Wenzel et al., 2011). Among salt-sensitive individuals, a low salt diet for 3 weeks decreases vascular stiffness (Al-Solaiman et al., 2009), suggesting that salt-induced inflammatory activation may have long-termeffects on vascular function. Importantly, recent findings suggest that vascular and renal inflammation in salt-sensitive hypertension may be initiated by oxidative stress (Barbaro et al., 2017). A recent clinical trial by Babcock et al. demonstrated that a reduction of dietary salt intake to 1,000 mg/dl for 10 days results in a significant decrease in sympathetic vascular transduction, suggesting that salt may contribute to vascular dysfunction through neuronal pathways as well (Babcock et al., 2019).

Recent evidence suggest that T cells also contribute to salt-sensitive hypertension through regulation of sodium chloride channels in the kidney. In a model of DOCA-salt mice, Liu et al. showed that CD8^+^ T cells directly contact the distal convoluted tubular and lead to the up-regulation of the thiazide-sensitive sodium-chloride-co-transporter (NCC) *via* ROS-induced Src kinase activation, resulting in the development of salt-sensitive hypertension (Liu et al., 2017). Recently, a study by Benson et al. found that the interaction between CD8^+^ T cells and NCC was mediated by IFN-γ-induced upregulation of MHC-I and PDL1 (programmed death-ligand 1) in distal convoluted cells, which induces NCC expression and increased sodium reabsorption. Abrogation of the IFN-γ response diminished renal T cell infiltration and salt-sensitive hypertension in DOCA-salt treated mice (Benson et al., 2022).

High salt-induced immune cell activation is also suggested to modulate blood pressure through alterations in the lymphatic capillary system. In rats, an increase in skin sodium accumulation with high salt diet activates tonicity-responsive enhancer binding protein (TonEBP) in APCs and leads to macrophage infiltration in tissue. TonEBP. a transcriptional regulator of the cellular response to hypertonic stress (Choi et al., 2020), further promotes the production of vascular endothelial growth factor-C (VEGF-C) by macrophages, resulting in increased nitric oxide synthase (eNOS) expression, hyperplasia of the lymphocapillary network and attenuation of salt-sensitive hypertension. In turn, treatment with VEGF-receptor blocker or macrophage depletion increases interstitial volume and salt-sensitive increase in blood pressure (Machnik et al., 2009).

## Oxidative stress and salt-sensitive hypertension

Oxidative stress is a well-known instigator of inflammation and appears to play a key role in the development of hypertension and related end-organ damage. Various studies suggest a link between high salt intake, exaggerated oxidative stress and hypertension (Rugale et al., 2003; Beltowski et al., 2004). A small clinical trial including 9 salt sensitive and 9 salt resistant subjects showed that the levels of urine F2-isoprostanes, a marker of oxidative stress, decrease with low salt diet in salt sensitive, but not salt resistant subjects (Al-Solaiman et al., 2009). High salt and fructose-fed rats demonstrate salt-sensitive hypertension along with decreased renal superoxide dismutase activity and ROS-induced activation of NF-κB (Dornas et al., 2017). High salt-fed rats demonstrate reduced antioxidant enzyme copper/zinc-dependent superoxide dismutase (SOD) compared to animals fed a normal diet, which is associated with higher vascular resistance (Durand and Lombard, 2013). Mice deficient in mitochondrial superoxide dismutase (MnSOD), demonstrate increased levels of oxidative and inflammatory markers and elevated blood pressure in response to salt. This is accompanied by increased urinary protein excretion, suggesting the development of salt-induced, oxidative stress-mediated renal dysfunction (Jin and Vaziri, 2014). Furthermore, cardiovascular benefits of HMG-CoA reductase inhibitors and angiotensin type 1 receptor antagonists (ARBs) are thoughts to be partially through attenuation of oxidative stress and the resulting improvement in endothelial function (Bayorh et al., 2007). In high salt-fed rat, simvastatin, a HMG-CoA reductase inhibitors and losartan, a ARB, were shown to decrease vascular and renal NADPH activity and ROS production (Bayorh et al., 2005). While the association between oxidative stress and salt-sensitive hypertension has been extensively studies, the exact mechanisms of how salt induces oxidative stress and immune activation have been shown only newly.

**FIGURE 1:**
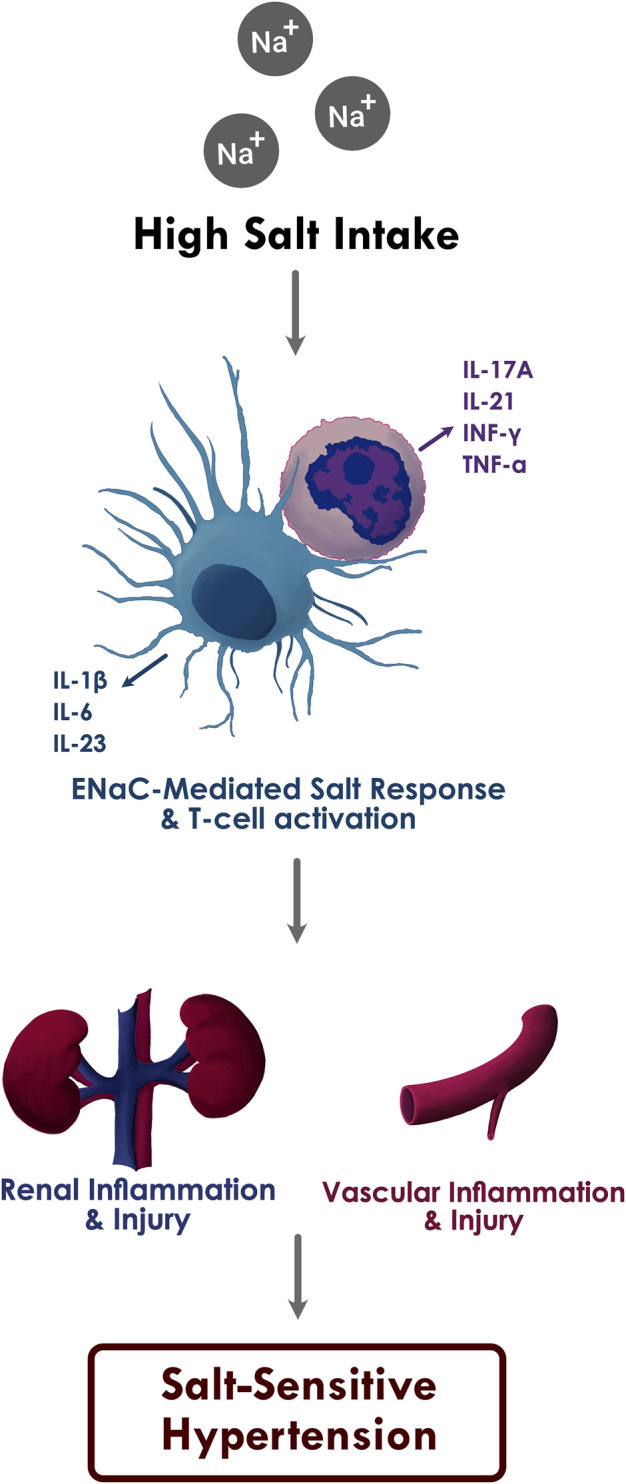
The proposed relationship between high sodium intake, inflammation, and salt sensitive hypertension.

In DOCA-salt treated mice models, we have shown that immune cell activation in salt-sensitive hypertension is led by the generation of isolevuglandins (IsoLGs; also called Isoketals or γ-ketoaldehydes), which are highly reactive oxidative products of arachnidonic acid metabolism (Wu et al., 2016). In high extracellular concentration, sodium enters APCs through the epithelial sodium channel (ENaC) and is exchanged with calcium (Ca^2+^) *via* the Na+/Ca2+ exchanger. Increased intracellular Ca^2+^ activates protein kinase C, which phosphorylates NADPH oxidase and leads to the formation of superoxide and IsoLGs. These highly unstable oxidative products adduct to proteins through the lysine residues and form IsoLG-protein adducts. These highly immunogenic protein adducts are subsequently presented on the MHC-II cell surface receptors and lead to T cell activation. These IsoLG-containing immune cells infiltrate the perivascular space and kidneys and secrete pro-inflammatory cytokines including IL-1β and IL-6 from the APCs and IFN-γ and IL-17A from the T cells, resulting in vascular and kidney dysfunction and ensuing hypertension (Figure 1) (Kirabo et al., 2014; Barbaro et al., 2017). Treatment with pharmacological scavengers of IsoLGs abolishes the salt-mediated immune cell activation and blood pressure response (Wu et al., 2016; Barbaro et al., 2017).

In addition to IsoLGs, heat shock proteins produced in response to oxidative stress may also drive immune cell activation in salt-sensitive hypertension. Pons et al. studied the role of heat shock protein 70 (HSP70) in immune activation in salt-sensitive rat model induced with l-NAME and subsequent high-salt diet. Immune tolerization to HSP molecule diminished renal inflammation and protected against the development of salt-sensitive hypertension (Pons et al., 2013). Hypertension correlates with an increased expression of heat shock protein 70 (HSP70) in lymphocytes (Kunes et al., 1992), which has been shown to trigger clonal expansion of CD4^+^ T cells in animal models (Pons et al., 2013).

Excessive formation of methylglyoxal (MGO), a highly reactive dicarbonyl metabolite that induces oxidative stress, has also been suggested to contribute to salt-sensitive hypertension (Ertuglu et al., 2021). Exogenous administration of MGO along with salt leads to elevated blood pressure compared to administration of salt alone in Sprague-Dawley rats (Guo et al., 2009). Vascular smooth muscle in hypertensive animals has increased MGO and ROS (Chang et al., 2005), and high fructose-induced increase in MGO levels has been associated with the development of hypertension (Wang et al., 2008), while the role of MGO in salt-sensitive hypertension specifically remains to be shown.

Salt-induced increase in ROS has also been suggested to contribute to hypertension through neuronal mechanisms. In rat models of hypertension, high salt intake has been shown to increase NADPH oxidase activity and ROS generation in the rostral ventrolateral medulla (Kishi et al., 2004), while injection of tempol, a potent radical scavenger, into the region resulted in reduction in blood pressure (Koga et al., 2008). The response to tempol was more pronounced during high-salt diet, suggesting that salt may mediate the oxidative stress seen in medulla (Koga et al., 2008).

Salt-sensitive hypertension disproportionally affects women. While the mechanisms of such sex discrepancy are yet unclear, emerging evidence suggest that inflammation and oxidative stress may play a role (Sahinoz et al., 2021b). In a recent study, Fernandes et al. demonstrated that high fat diet promotes hypertensive renal inflammation and injury and correlated with blood pressure only in male, but not female, Dahl salt-sensitive rats. Furthermore, female Dahl rats had less renal T cell and macrophage infiltration and higher renal regulatory T-cells cell ratio compared to males regardless of diet (Fernandes et al., 2018). Another study by Belanger et al. showed that female Sprague Dawley rats had greater number of T regulatory cells, which was protective against DOCA-salt induced hypertension and renal damage. Treatment with anti-CD25 to decrease regulatory T cells abolished the sex differences in DOCA-salt induced blood pressure response (Belanger et al., 2020). Studies in preeclampsia, a pregnancy-specific syndrome characterized by hypertension and proteinuria, have found that monocytes from preeclamptic women have higher expression of NLRP3 (NOD-like receptor family pyrin domain containing 3) inflammasome, caspase-1, and IL-1β compared with normotensive pregnant women, suggesting that inflammasome-mediated inflammation may be implicated in sex-specific hypertensive responses (Matias et al., 2015).

## Inflammasome activation as a driver of salt-sensitive hypertension

Emerging evidence suggests that inflammasome, a major component of innate immune response, is a driver of inflammation in hypertension (De Miguel et al., 2021). Inflammasomes are intracellular sensors of pathogen-associated molecular patterns (PAMPs) and endogenous host-derived damage-associated molecular patterns (DAMPs). Among several types of inflammasomes NLR family CARD domain-containing protein 4 (NLRC4), NLRP6 and NLRP9, NLRP3 is most studied inflammasome in hypertension. NLRP3, a member of the nucleotide-binding oligomerization domain leucine-rich repeat (NLR) PRR family, is composed of apoptosis-associated speck-like protein and a caspase activating recruitment domain (ASC) and can be activated in response to a wide range of molecular and cellular events through the canonical and non-canonical pathways. In the canonical pathway, a priming signal that can be provided by Toll-like receptors (TLRs), the nucleotide-binding oligomerization domain (NOD) 1 and 2 or cytokine receptors, activates the nuclear factor kappa B (NF-κB), which in turn upregulates the expression of NLRP3 and pro-IL-1β (Kelley et al., 2019). This initial step is crucial since the cellular concentrations of NLRP3 and pro-IL-1β are insufficient to orchestrate inflammasome activation under normal conditions (Bauernfeind et al., 2009). Following the priming, the second (activation) signal can be provided by a number of stimuli including ATP, pathogen associated RNA, bacterial or fungal toxins and components as well cellular signals including ion influx, reactive oxygen species, viral RNA, toxins and mitochondrial and lysosomal damage, most of which trigger potassium efflux, a common trigger for NLRP3 activation (Muñoz-Planillo et al., 2013). NEK7, a member of the family of mammalian NIMA-related kinases (NEK proteins), is an NLRP3-binding protein that mediates NLRP3 assembly in response to the secondary signal, leading to the activation of caspase-1 and cleavage of pro-IL-1β. Activation of caspase-1 also leads to the cleavage of gasdermin (GSDMD), formation of membrane pores and resulting pyroptosis (He et al., 2016; Shi et al., 2016). The non-canonical pathway is activated by LPS of Gram-negative bacteria and is mediated by induction of caspase-11 (Kayagaki et al., 2011). Caspase-11 activation droves potassium efflux that activates NLRP3 inflammasome while also cleaving GSDMD that results in pyroptosis (He et al., 2015; Kayagaki et al., 2015; Chen et al., 2019).

NLRP3 activation results in caspase-1 activation and subsequent secretion of IL-1β and IL-18 (Chen and Nunez, 2010; Van Tassell et al., 2013; Lamkanfi and Dixit, 2014), the plasma levels of which are consistently high in patients with hypertension (Dorffel et al., 1999; Li et al., 2005; Rabkin, 2009). The levels of these pro-inflammatory cytokines also correlate with vascular and renal dysfunction in this population (De Miguel et al., 2021). The inductin of pyroptosis by NLRP3 activation, which is a lytic, pro-inflammatory type of cell death (Wang et al., 2019), further induces the release of more IL-1β, IL-18 and other pro-inflammatory intracellular contents, and therefore aggravates the inflammatory response. NLRC4 is capable of forming inflammasome complexes has been identified to play a role in hypertension. For instance, upregulation of NLRC4 gene in immune cells plays a role in age-related hypertension and vascular dysfunction (Furman et al., 2017). However, *in vitro* studies of elevated sodium concentration indicate that only NLRP3 may be the onlysalt-responsive inflammasome (Prager et al., 2016; Pitzer et al., 2022), while other inflammasomes such absent in melanoma 2 (AIM2) have been implicated in aldosterone-induced renal inflammation and damage (Wu et al., 2022).

Genetic mutations of the NLRP3 gene have been shown to be associated with the development of hypertension. Among a 50-year old Finnish cohort of 769 patients, single nucleotide polymorphism in NLRP3 gene, rs7512998, has been found to associate with higher blood pressure. Furthermore, the patients with the polymorphism had greater blood pressure in a 5-year follow-up period (Kunnas et al., 2015). A variable number of tandem repeat polymorphism in CIAS1 gene that encodes NLRP3 has also been associated with increased risk of hypertension (Omi et al., 2006; De Miguel et al., 2015). Additionally, NLRP3 single nucleotide polymorphism variant rs10754558 (C>G) plays an important role in the severity of COVID-19 in elderly males with hypertension (Maes et al., 2022). Polymorphisms in the IL-1β gene have also been associated with elevated blood pressure (Lin and Morris, 2002; Huang et al., 2004; De Miguel et al., 2021).

Animal models of salt-sensitive hypertension are found to have increased mRNA expression of NLRP3 subunits along with increased protein levels of active caspase-1 and mature IL-1β in the kidney (Krishnan et al., 2016). ASC deficient mice and mice treated with MCC950, a novel NLRP3 inflammasome inhibitor, are protected from salt-induced renal inflammation, fibrosis and hypertension (Krishnan et al., 2016; Krishnan et al., 2019). Corroborating with these findings, NLRP3 activation has been associated with kidney injury in various disease models (Shahzad et al., 2015; Haque et al., 2016; Chi et al., 2020). Furthermore, renal tubular cells from DOCA-salt treated mice demonstrate increased expression of IL-18, while IL-18 deficient mice were protected from increased blood pressure and renal fibrosis (Thomas et al., 2021). Inhibition of NLRP3 inflammasome activity with MCC950 ameliorates hypertension, renal inflammation and fibrosis in DOCA-salt treated mice (Krishnan et al., 2019). Nevertheless, Ling et al. did not find a beneficial effect of IL-1 receptor blockage with anakinra on renal inflammation in DOCA-salt treated mice despite a substantial antihypertensive effect (Ling et al., 2017), suggesting that anakinra may modulate blood pressure through extra-renal mechanisms.

NLRP3 inflammasome has also been implicated in the development of vascular dysfunction in hypertension (De Miguel et al., 2021). NLRP3 inflammasome activation through IL-1 receptor has been shown to be crucial in the development of aldosterone-induced vascular dysfunction. Studies by Bruder et al. have found that mice lacking IL-1 receptor or the NLRP3 inflammasome components were protected from aldosterone-induced vascular damage, and the effects of NLRP3 inflammasome were mediated by immune cell activation (Bruder-Nascimento et al., 2016). Hyperhomocysteinemia, a risk factor for the development of hypertension and its complications (Skeete and DiPette, 2017), has been shown to induce NLRP3 inflammasome assembly, caspase-1 activation and pyroptosis in endothelial cells, while mice deficient in caspase-1 or NLRP3 are protected (Xi et al., 2016). NLRP3-deficient mice were also shown to be protected from salt-induced endothelial dysfunction (Fu et al., 2018). In addition, NLRP3 inflammasome also modulates nitric oxide signaling, which is disrupted in salt-sensitive hypertension (Sogawa et al., 2018). Pharmacologic inhibition of NLRP3 expression in aortic endothelial cells also prevents the decrease in endothelial nitric oxide synthase expression induced by high salt (Fu et al., 2018). These studies suggest that NLRP3 contributes to salt-mediated vascular injury.

Nuclear factor kappa B (NF-κB) activation upregulates the expression of NLRP3 and pro-IL-1β preparing the inflammasome complex for activation by subsequent stimulant (Wang et al., 2019). This process is referred to as priming where NF-κB plays a critical role. Hypertension has been characterized by elevated levels of NF-κB in tissue and inflammatory cells (De Miguel et al., 2021). The inhibition of NF-κB in animal models of hypertension decreases blood pressure and protects from end-organ damage (Rodríguez-Iturbe et al., 2005; Zambom et al., 2019). In rat models of salt-sensitive hypertension, inhibition of NF-κB with pyrrolidine dithiocarbamate (PDTC) improves vasodilation and blood pressure (Zhou et al., 2010). Additionally, blocking NF-κB activity in the hypothalamic paraventricular nucleus downregulated NLRP3 and IL-1β, promoting upregulation of anti-inflammatory cytokines and delayed progression of salt-sensitive hypertension in Dahl salt-sensitive rats (Qi et al., 2016).

**FIGURE 2:**
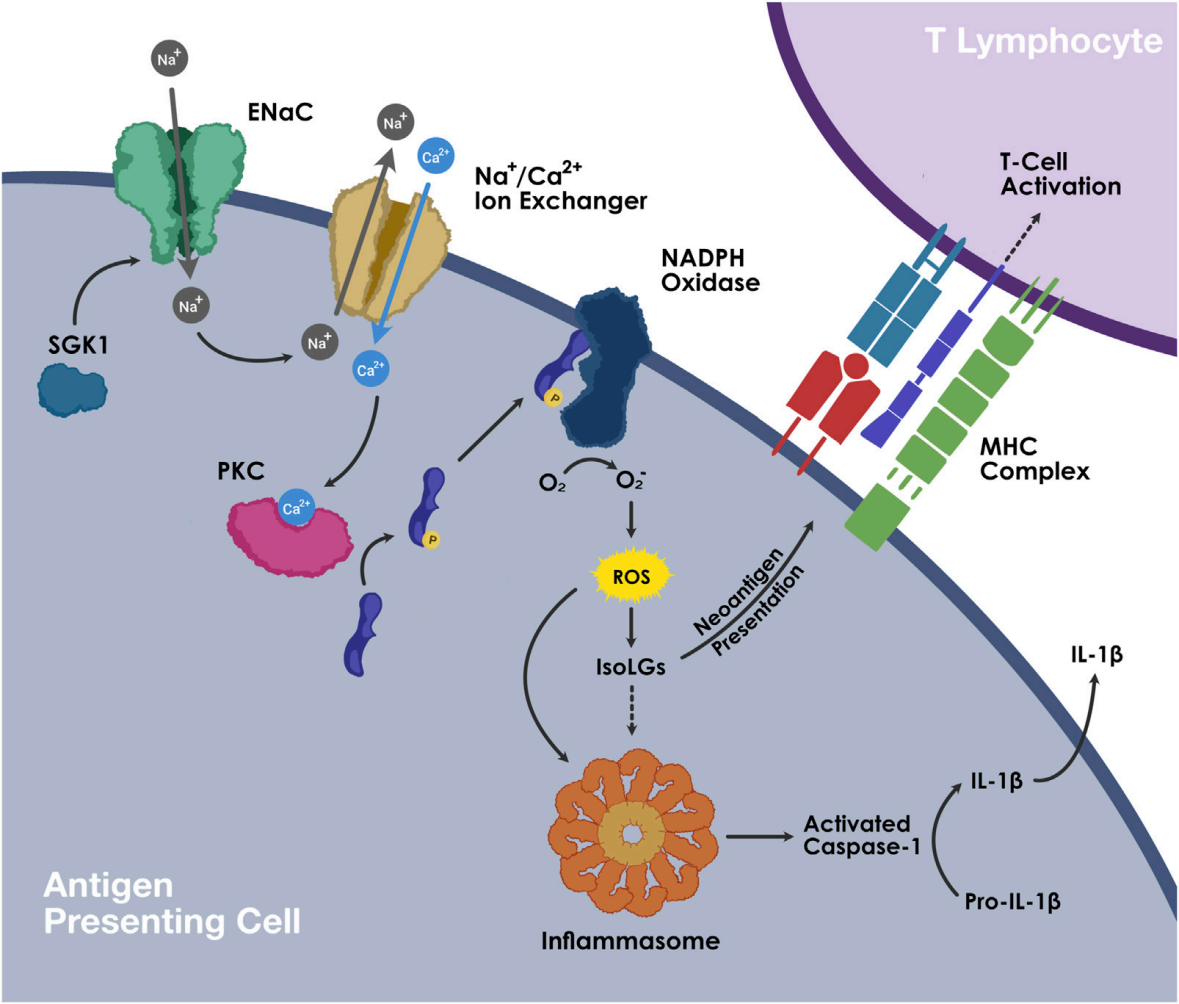
The mechanism of ENaC and IsoLG-dependent NLRP3 inflammasome activation in APCs.

Oxidative stress is a known trigger of NLRP3 inflammasome activation (Abais et al., 2014; Cui et al., 2014; Banoth and Cassel, 2018). In very recent studies, we demonstrated that NLRP3 inflammasome activation plays a crucial role in ENaC- and IsoLG-dependent APC activation and the ensuing inflammation in salt-sensitive hypertension (Pitzer et al., 2022). In a cohort phenotyped for salt sensitivity using the inpatient protocol of salt loading and depletion, cell hashing, and cellular indexing of transcriptomes and epitopes of peripheral blood mononuclear cells has shown that NLRP3 inflammasome expression and IL-1β mirrored changes in blood pressure following salt depletion, indicating that dietary sodium intake acutely regulates monocyte NLRP3 activity in humans. *In vitro* exposure of human monocytes collected from another cohort to high or normal sodium demonstrated that high sodium exposure upregulated the expression of caspase-1, IL-1β and IL-18. Furthermore, DCs and monocytes from salt-sensitive mice exhibit increased intracellular IL-1β production in response to high salt exposure. Inhibition of NLRP3 inflammasome with MCC950 and caspase-1 inhibitor YVAD abrogated this response, showing that high sodium–induced IL-1β formation is downstream of NLRP3 inflammasome activation in APC (Pitzer et al., 2022). Importantly, splenic DCs and monocytes from salt-sensitive mice fed with 4-week high salt diet demonstrate increased accumulation of NLRP3, IL-1β along with IsoLG-protein adducts. Co-administration of MCC950 with the high salt diet attenuates salt-induced increase in APC NLRP3, IL-1β and IsoLG-adducts as well as blood pressure. We further found that high salt-induced activation of NLRP3 inflammasome is ENaC- and IsoLG-dependent. Moreover, adaptive transfer of CD11c+ myeloid cells induced salt-sensitive blood pressure elevation in NLRP3 knockout mice (Pitzer et al., 2022). These findings show that NLRP3 inflammasome activation contributes to ENaC-mediated, IsoLG-induced salt sensitivity of blood pressure (Figure 2).

## Perspectives

Salt-sensitivity of blood pressure is a strong risk factor for cardiovascular morbidity and mortality, and therefore a major target in reducing poor outcomes especially in the hypertensive population. While the precise pathogenesis remains incompletely understood, the roles of innate and adaptive immunity and oxidative stress in the mechanism of salt-sensitivity are well established. Emerging evidence demonstrates that inflammasome activation and pyroptosis are important mediators of inflammation and cardiorenal damage in hypertension. Recently, ENaC-dependent, oxidative stress-mediated inflammasome activation in APCs is found to drive salt-sensitive hypertension, introducing a potential target in this population. While targeting oxidative stress and inflammasome activation in immune cells is a promising therapeutic approach for the management of salt-sensitive hypertension, no human studies have yet shown a benefit of such treatment. The Canakinumab Anti-Inflammatory Thrombosis Outcome Study (CANTOS) found that 3 months treatment with canakinumab, an anti-IL-1β antibody, significantly decreases recurrent cardiovascular events in patients with previous myocardial infarction and elevated high-sensitivity C-reactive protein levels during a follow-up period of 3.7 years, while no effect was observed in blood pressure or all-cause mortality. Canakinumab treatment was associated with significantly higher incidence of fatal infections (Ridker et al., 2017).

Although substantial evidence established oxidative stress as a major driver of cardiometabolic disease, clinical trials to date have failed to show efficacy of anti-oxidants (Stephens et al., 1996; Rapola et al., 1997; Virtamo et al., 1998; Yusuf et al., 2000; Lonn et al., 2001; Heart Protection Study Collaborative Group, 2002; Vivekananthan et al., 2003; Lonn et al., 2005; Harrison and Gongora, 2009). While several factors, including the use of sub-optimal doses of anti-oxidants below that is required to significantly alter lipid peroxidation (Roberts et al., 2007), very high doses of anti-oxidants have been paradoxically associated with increased cardiovascular mortality (Widder and Harrison, 2005). Since ROS have vital physiological roles besides harmful effects, their complete elimination may not be possible or desirable. Thus, targeting downstream products of oxidative stress that play key roles in the development of disease, such as IsoLGs, could have significantly fewer adverse effects and provide greater protection.

Future human studies are essential to assess the utility of immune-targeted therapies in salt-sensitive hypertension. However, the current methods for assessment of salt-sensitivity in humans include cumbersome protocols of salt loading and depletion, significantly limiting the feasibility of large-scale clinical studies. The recent findings of the role of oxidative stress and inflammasome activation do not only expand our understanding of the pathogenesis of salt-sensitivity, but also suggest potential diagnostic biomarkers. Improving our understanding of pathways leading to salt sensitivity and related end-organ damage is crucial for the discovery of diagnostic tools that can be used in the clinic as well as therapies targeted specifically for this high-risk population.

Increased ENaC-mediated sodium entry into the APCs through high salt intake leads to oxidative stress in APCs, which in turn activates T cells and results in the production of pro-inflammatory cytokines IL-1β, IL-6, and IL-23 from APCs and IL-17A, IL-21, IFN-γ, and TNF-α from T cells. Subsequent inflammatory response and immune cell infiltration causes renal and vascular damage and salt sensitive hypertension.

At high concentrations of extracellular sodium, sodium enters APCs *via* amiloride-sensitive ENaC and leads to intracellular influx of calcium *via* the sodium-calcium ion exchanger. Increased calcium stimulates protein kinase-C, which in turn phosphorylates p47*phox*, activating NADPH oxidase. ROS production by NADPH oxidase leads to the generation of isolevuglandins (IsoLGs). ROS and IsoLGs activate NLRP3 inflammasome. Activated caspase-1 cleaves pro-IL-1β into mature IL-1β. . IsoLGs react with lysine residues on proteins and form IsoLG-adducts, which are subsequently presented on MHCs and promote T cell activation.
